# Recent Advances in HIV-1 Gag Inhibitor Design and Development

**DOI:** 10.3390/molecules25071687

**Published:** 2020-04-07

**Authors:** Alexej Dick, Simon Cocklin

**Affiliations:** Department of Biochemistry & Molecular Biology, Drexel University College of Medicine, Rooms 10307, 10309, and 10315, 245 North 15th Street, Philadelphia, PA 19102, USA; ad3474@drexel.edu

**Keywords:** HIV-1 Gag polyprotein, antiretrovirals, matrix protein, capsid protein, nucleocapsid protein, p6 protein

## Abstract

Acquired Immune Deficiency Syndrome (AIDS) treatment with combination antiretroviral therapy (cART) has improved the life quality of many patients since its implementation. However, resistance mutations and the accumulation of severe side effects associated with cART remain enormous challenges that need to be addressed with the continual design and redesign of anti-HIV drugs. In this review, we focus on the importance of the HIV-1 Gag polyprotein as the master coordinator of HIV-1 assembly and maturation and as an emerging drug target. Due to its multiple roles in the HIV-1 life cycle, the individual Gag domains are attractive but also challenging targets for inhibitor design. However, recent encouraging developments in targeting the Gag domains such as the capsid protein with highly potent and potentially long-acting inhibitors, as well as the exploration and successful targeting of challenging HIV-1 proteins such as the matrix protein, have demonstrated the therapeutic viability of this important protein. Such Gag-directed inhibitors have great potential for combating the AIDS pandemic and to be useful tools to dissect HIV-1 biology.

## 1. Introduction and Current Status of Antiretroviral Therapies

AIDS (Acquired Immune Deficiency Syndrome) is a global epidemic caused by HIV (human immune-deficient virus) infection [1]. At the end of 2018, 37.9 million people were living with HIV worldwide, with 1.7 million newly infected and 770,000 AIDS-related deaths [2]. By the end of 2017, the Food and Drug Administration (FDA) has approved 43 anti-retroviral drugs for clinical use [3]. With the introduction of combination antiretroviral therapy (cART) in 1996, AIDS-related deaths have declined dramatically. cART is a three-component treatment, composed of drugs with at least two independent mechanisms of action. Typical combinations are usually two nucleoside/nucleotide reverse transcriptase inhibitors (NRTIs) combined with a protease inhibitor (PI), a non-nucleoside reverse transcriptase inhibitor (NNRTI) or an integrase strand transfer inhibitor (INSTIs) [4,5]. Patients on cART display decreased virus loads and increased CD4^+^ cell numbers that have prolonged patient survival and led to the establishment of AIDS as a manageable chronic disease. However, eradication of HIV is not possible via cART due to a pool of latently infected CD4^+^ T cells in the acute early infection phase, and if the dosing regimen is not followed accurately, viral load rebounds can occur accompanied by viral resistance [4,6]. Long-term cART therapy also leads to side effects and age-related comorbidities such as diabetes, cardiovascular, renal, and bone diseases and can result in a reduced life expectancy of HIV-1 infected patients [7,8]. This highlights the continued need for new antiretroviral drugs with low cytotoxicity, long-acting formulations, and new targets in the HIV-1 replication cycle. One such emerging therapeutic target is the HIV-1 Gag protein, which is the master regulator of co-factor packaging, assembly, and release of the immature virion.

In this review, we describe topologically (from the N-terminal, matrix protein to the C-terminal, p6 domain) the importance of targeting the HIV-1 Gag polyprotein and its component domains for the development of novel antivirals. In addition to current Gag-targeted inhibitors, we highlight some of the new developments for each Gag domain and speculate, based on these recent findings, on possible future antiviral designs.

## 2. The Gag Polyprotein and Its Role in the HIV-1 Replication Cycle

**Figure 1:**
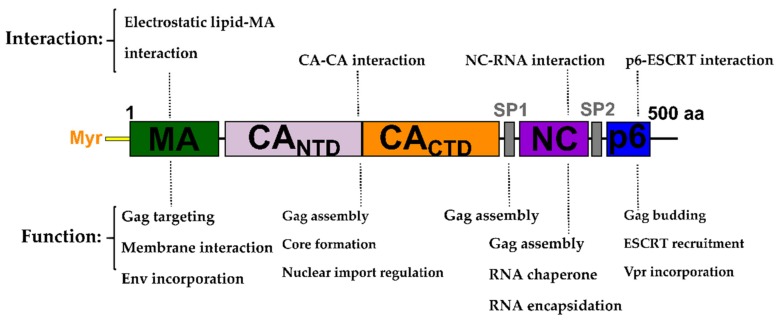
Domain architecture of the Gag precursor polyprotein. The function (**bottom**) and type of interaction (**top**) are highlighted. Matrix (MA) is responsible for Gag targeting to cholesterol-enriched lipid rafts for virus budding at the plasma membrane (PM) and incorporation of Env. Capsid (CA) is crucial for Gag assembly and the formation of the conical core structure. Interaction with host cell factors such as cyclophilin A (CypA) or transportins regulates the nuclear import of the pre-integration complex. SP1 is involved in Gag assembly. Nucleocapsid (NC) is involved in Gag assembly and, with its two zinc fingers, binds to RNA and exerts RNA chaperone activity. P6 is involved in the recruitment of the endosomal sorting complex required for transport (ESCRT) for virus egress and in Vpr incorporation.

In the late stage of the HIV-1 replication cycle, the assembly of newly synthesized virions and the incorporation of viral and cellular components need to be orchestrated and directed to the plasma membrane. The HIV-1 Gag polyprotein is the master coordinator of the assembly of viral particles. The HIV-1 Gag polyprotein is translated in the host cell cytosol as a 55 kDa protein, comprised of several domains that are cleaved into individual proteins post-viral budding. Gag contains the matrix (MA), capsid (CA), and nucleocapsid (NC) proteins, in addition to a small p6 domain and two spacer peptides (Figure 1) [9].

**Figure 2:**
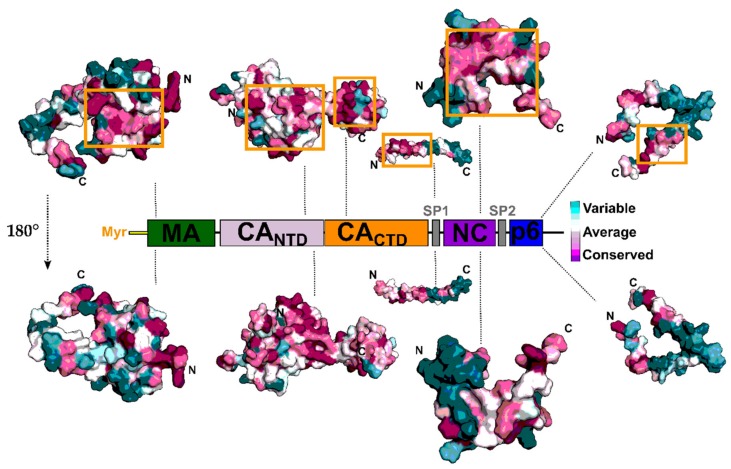
Surface conservation of the HIV-1 Gag polyprotein and inhibitor target sites. Alignment of 9547 HIV-1 Gag sequences were retrieved from the HIV Los Alamos database (www.hiv.lanl.gov). Sequences were aligned against the HxBc2 reference. Conservation analysis was performed using the ConSurf server [10]. Structures of the HIV-1 Gag domains with PDB entries: matrix, 2H3Z; capsid, 6ES8; sp2, 1U57; nucleocapsid, 2M3Z; p6, 2C55. Orange boxes represent target sites for inhibitor binding highlighted in this review. Low conservation in light cyan to high conservation in dark purple.Gag’s constituent proteins act at different points in the viral life cycle. MA binds specifically to phosphoinositide 4,5-bisphosphate (PI [4,5]P_2_) and specific phospholipids on the plasma membrane, triggering the exposure of an attached myristoyl (myr) chain and directing Gag to the membrane. This membrane interaction is required for the correct incorporation of the viral envelope protein (Env) into the budding virus [11,12,13,14]. In the late stages of the replication cycle, CA is responsible for the assembly of Gag at the plasma membrane by providing intermolecular contact sites for Gag oligomerization at the plasma membrane [15,16]. In the early stages of replication, CA disassembly regulates the process of reverse transcription, and its engagement of cellular transportins and nuclear pore components facilitate the import of the viral pre-integration complex into the nucleus, where integration takes place [17]. NC functions as a nucleic acid chaperone at multiple steps in the HIV-1 replication cycle, and it’s overall positively charged character and two zinc-finger motifs allow it to interact with viral genomic RNA via the RNA packaging signal and thereby facilitate virion assembly [18,19,20,21]. Finally, the p6 domain (late domain) recruits the endosomal sorting complex required for transport (ESCRT) machinery to promote virus budding and final release [22]. Two spacer peptides (SP1 and SP2) flanking the NC domain regulate the kinetics of Gag maturation, and SP1 also provides, as part of the C-terminus of CA, another Gag-Gag multimerization interface [23,24]. Because Gag functions in so many different aspects of viral infection and replication, Gag inhibitors have the potential to exert their effects in both early and late stages of the replication cycle, making this polyprotein a particularly attractive target for the development of new therapeutics.

These individual proteins play multiple essential roles in the life cycle of the virus by interacting with host dependency factors. As such, the regions on the proteins responsible for these interactions and processes display high conservation, making them ideal areas to target using small molecules or peptides. Accordingly, Gag has recently emerged as an attractive therapeutic target. The surface conservation of the individual Gag domains is highlighted in Figure 2.

## 3. HIV-1 Protease and Maturation Inhibitors

Maturation is an essential step in the HIV-1 replication cycle and results in the release of the individual Gag domains, which perform multiple crucial functions. Maturation inhibitors represent a novel class of antiretrovirals targeting the CA-SP1 cleavage site. HIV-1 maturation can be divided into two steps: (1) assembly and release at the PM, and (2) proteolytic cleavage by the viral protease (PR). During assembly and budding, Pr55Gag (immature Gag precursor) hexamerizes at the plasma membrane. Through orchestrated interactions, primarily with the ESCRT proteins, virions are initially released as immature virus particles. Immature virus particles are non-infectious and have a hexameric Gag lattice without the characteristic conical core architecture [15,25]. Proteolytic processing of Gag and Gag-Pol polyprotein (Gag precursor encoding viral protease, integrase, RNase H, and reverse transcriptase) by the viral protease (PR) is a complex multilayer process with multiple cleavage sites and substrates. One of many cleavage sites occurs between the C-terminal portion of CA and SP1 junction and triggers a conformational switch that destabilizes the immature Gag and the formation of the mature conical core [26,27]. Maturation inhibitors target this cleavage site, resulting in the accumulation of CA-SP1 precursor, which in turn causes an infectivity loss. Inhibition of maturation can be subdivided into (1) inhibition of the viral PRs catalytic activity as PR-Inhibitors and (2) inhibition of Gag cleavage by maturation inhibitor (MI) [28].

**Figure 3:**
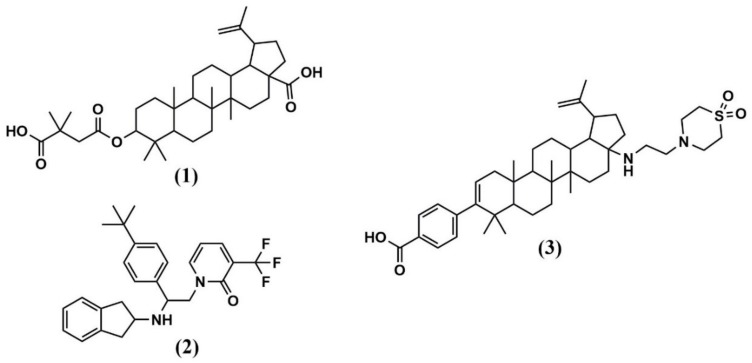
Structure of maturation inhibitors (MI) discussed in this review. (**1**) Bevirimat (BVM); (**2**) PF-46396; (**3**) GSK3532795.

The first identified maturation inhibitor was Bevirimat (BVM) or also known as 3-O-(3′,3′-dimethylsuccinyl) betulinic acid, PA-457, or MPC-4326 (**1**) (Figure 3 and Table 1). BVM caused abnormal virion morphology and inhibition of viral replication with an IC_50_ of around 10 nM [29]. Resistance mutation generation confirmed the CA-SP1 junction site as the target binding site [30]. Additionally, electron cryomicroscopy and electron diffraction of thin 3D microcrystals (MicroED) provided insight into the mechanism of action of BVM and revealed that one BVM molecule binds and stabilizes the six-helix bundle in a CA hexamer via both electrostatic and hydrophobic interactions [31]. Binding in the center of the six-helix bundle stabilizes the hexameric immature Gag lattice and ultimately prevents the final cleavage event in Gag processing, the separation of CA from its spacer peptide (SP1).

Despite successful phase I and phase II clinical trials, the phase IIb trial failed due to the non-responsiveness of a large patient group because of the rapid emergence of resistance mutations found in the CA-SP1 site of those patients. Interestingly, a single nucleotide polymorphism (SNP) at Val7 to Ala in the SP1 region is present in the consensus sequences of subtype C, D, F, and G [32,33]. As the Subtype C virus accounts for approximately 50% of the HIV-1 infections worldwide, the presence of these SNPs resulted in discontinuation of BVM as a clinically viable antiretroviral.

Further explorations identified a second compound, PF-46396 (**2**), although with a lower potency compared to BVM [34] (Figure 3 and Table 1). PF-46396 is structurally different from BVM, but both target Gag cleavage at the CA-SP1 site, as indicated by in vitro resistance mutation development [34,35]. Besides targeting a similar binding site, PF-46396 induced resistance mutations at different locations, suggesting a different binding mode as compared to BVM. PF-46396 resistance mutations were identified in three regions of Gag: around the CA-SP1 cleavage site similar to BVM but additionally also, at CA amino acid 201 (I201V), and in the CA major homology region (MHR, G156E, P157S, P160L), indicating implications for Gag assembly, release and virus replication [35]. However, these two compounds, BVM and PF-46396, as first in class maturation inhibitors, highlight the feasibility of this strategy for antiviral development and warranted further study and optimization. Generating a targeted library of betulinic acid derivatives, and screening against a panel of engineered reporter viruses with site-directed alterations in Gag that reduced susceptibility for BVM resulted in the identification of a second-generation MI, GSK3532795 (**3**) (formerly known as BMS-955176) [36] (Figure 3 and Table 1). This second-generation MI displayed promising potency against a panel of subtype B isolates (EC_50_ of 21 nM) and had a significantly improved preclinical profile as compared to BVM. Time-of-addition studies also confirmed inhibition of HIV-1 replication in a late-stage by inhibiting CA-SP1 cleavage.

Freed and colleagues further demonstrated that this second-generation inhibitor could partly overcome the resistance problem of BVM with markedly higher potency and activity against subtype B and the world’s dominant circulating subtype C [37,38,39,40,41,42,43]. This encouraging study leads to the transition of these second-generation inhibitors into the phase II trial with a promising outlook for patients with a developed resistance to currently available therapies. A recent randomized phase IIb study showed that GSK3532795 could reduce plasma HIV-1 RNA below 40 copies/mL at Week 24 [44]. Despite these significant efficacy rates, the clinical development of GSK3532795 was terminated due to high rates of adverse gastrointestinal events, and frequency of treatment-emergent nucleoside reverse transcriptase inhibitor (NRTI) resistance. However, the antiviral response rates and immunologic reconstitution for GSK3532795, together with a novel mechanism of action is promising and support the continued development of MI inhibitors as anti-HIV-1 agents. The microED structure of BVM in complex with HIV-1 CA provides additional insights for rational drug design on MIs [31].

## 4. Matrix (MA, p17)

**Figure 4:**
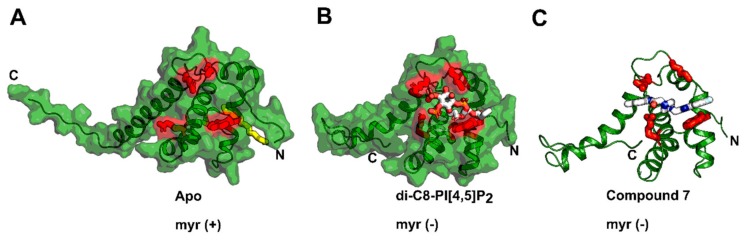
MA inhibitors targeting the PI[4,5]P_2_ binding site. (**A**) The NMR structure of MA with its myristic acid (in yellow) buried in a hydrophobic groove at the N-terminus (PDB code: 2H3I). (**B**) The NMR structure of MA bound to di-C4-PI[4,5]P_2_ displacing the myristic acid (PDB code: 2H3Z). (**C**) Docking model of compound 7 bound to MA and displacing di-C4-PI[4,5]P_2._ In red are the residues highlighted that are involved in di-C4-PI[4,5]P_2_ binding.

The HIV-1 matrix (MA) protein is a key player in virus assembly. It is encoded as the N-terminal portion of the Gag polyprotein, and like the other Gag components (CA, NC, and p6), it displays high conservation in functional regions between HIV-1 subtypes, making it a very attractive target for intervention. This small, multifunctional protein is responsible for directing the viral and cellular components to the site of assembly and regulating the incorporation of the envelope (Env) glycoproteins into the budding virus [12,45,46]. A buried hydrophobic myristoyl group (myr) and a basic patch at the N-terminus of the MA protein are crucial for the association of the MA protein to the PM. The basic region of MA has been demonstrated to specifically interact with PI[4,5]P_2_ and other PM lipids such as phosphatidylserine, phosphatidylcholine, and phosphatidylethanolamine [47]. The basic patch also has nucleic acid binding properties, and it is thought that the interplay between lipid and RNA binding is critical for a specific interaction with only the plasma membrane [11,48]. Host cell proteins such as Arf (ADP ribosylation factor) and GGA (Golgi-localized γ-ear containing Arf-binding protein) have been demonstrated to facilitate the trafficking of Gag/MA to the PM [49]. Binding to PI[4,5]P_2_ facilitates the exposure of the buried/folded myr moiety and promotes oligomerization at the PM (Figure 4A,B) [12,50,51]. Myristate exposure is believed to be triggered by an allosteric mechanism, by which PI[4,5]P_2_ binding induces conformational changes at the N-terminal β-hairpin and helix α-1. This conformational alteration repositions hydrophobic residues and displays the myristyl group and stabilizes the myr (exposed) state of the MA protein. Early structural work revealed that HIV-1 MA forms crystallographic trimers, and recently identified trimerization interface mutants have been demonstrated to interfere with Env incorporation, suggesting a biological relevance of the MA trimers [52,53,54,55]. In agreement with these observations, the HIV-1 MA and MA-CA fusion proteins organize as hexamers of trimers predominantly at lipid rafts (PI[4,5]P_2_/Cholesterol containing membranes) [56]. Those hexameric structures also have implications for Env incorporation in immature HIV-1 virions [54,55,57,58,59]. HIV-1 Env incorporation into immature virions is believed to be directed by the long cytoplasmic tail (CT) of gp41 and steric trapping within these MA hexamers of trimers [13,60,61,62,63,64,65]. This process is highly regulated, considering the low number of Env incorporated into released particles (7–14 trimers) [66].

Because of its fundamental roles in virion assembly and the high degree of conservation of its PI[4,5]P_2_/nucleic acid binding site, the HIV-1 MA protein has emerged as an attractive, antiviral target [11,48,67,68].

Besides its high conservation, the highly basic PI[4,5]P_2_/RNA binding site, which is currently the main target site for inhibitor design, represents major challenges for small molecule targeting, such as its shallow architecture and a high degree of entropy based on positively charged residues, mainly lysines and arginines.

**Figure 5:**
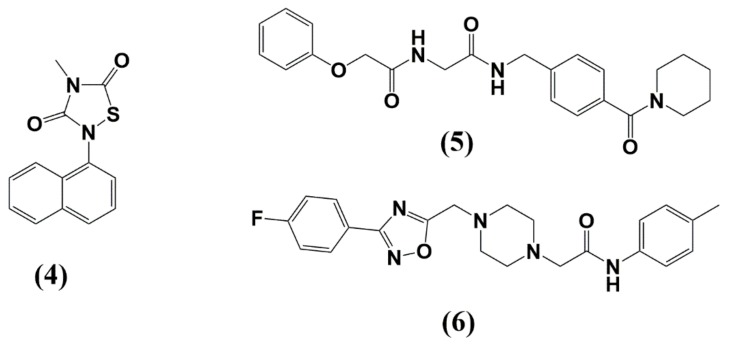
Structure of MA inhibitors discussed in this review. (**4**) TD2; (**5**) compound 7; (**6**) compound 14.

Nevertheless, compounds have been described targeting the nuclear localization signal (NLS) of MA [69] or the MA-RNA interaction [70]. Thiadiazolane based compounds (**4**) that target the MA-RNA interaction could inhibit HIV-1 replication in cell cultures; however, inhibition was associated with significant levels of toxicity (Figure 5 and Table 1) [70]. Using virtual and surface plasmon resonance (SPR)-based screening, Zentner et al. discovered the first inhibitors targeting the PI[4,5]P_2_ binding site in MA without cytotoxic effects [71,72]. The best Compound 7 (**5**) displayed cross-clade anti-HIV activity with IC_50_ values of 7.5-–15.6 µM for group M isolates (Figure 4C and Figure 5, and Table 1).

Site-directed mutagenesis and PI[4,5]P_2_ SPR-based competition assay confirmed the PI[4,5]P_2_ binding site as the interaction site for Compound 7, and accordingly, mutations such as L21A and T81A in a pseudotyped virus lost susceptibility to the compounds tested. This work first demonstrated the feasibility of targeting the MA protein with small drug-like molecules. Unfortunately, the initial chemotypes identified were subject to activity cliffs and have been abandoned (Cocklin et al., Unpublished). Despite this initial setback, Cocklin et al. have continued to pursue the identification of MA-targeted inhibitors and have recently discovered a promising new chemotype (Cocklin et al., Unpublished). Work is actively ongoing in optimizing the affinity/potency of this new chemotype, in the hopes that it may serve as a template to a new class of anti-HIV-1 therapeutics.

**Figure 6:**
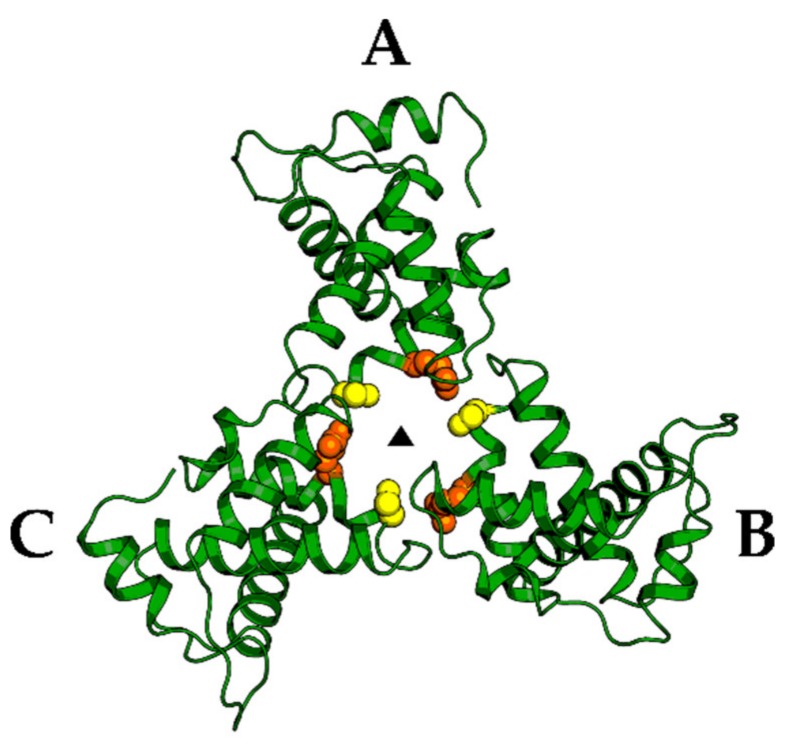
HIV-1 MA trimerization is essential for HIV-1 Env incorporation. Residues involved in the trimerization interface Gln62 in orange and Ser66 in yellow are putative target sites for novel inhibitor designs. PDB code: 1HIW.

Despite the highlighted progress targeting the challenging PI[4,5]P_2_/RNA binding site, new sites of attack are highly desirable. One such point of attack could be the involvement of MA in Env incorporation. As mentioned above, MA trimerization and hexamerization of these trimers are crucial for Env incorporation. Mutagenesis of trimer interface residues clearly showed a correlation between loss of MA trimerization by introducing trimer disrupting mutations and loss of Env incorporation in the context of the virus [54,55,73]. Consequently, the stabilization of the MA trimers by introducing a glutaraldehyde crosslinking approach at Ser66 and Gln62 or introducing a Gln62Arg mutation in the trimer interface (Gln62 represented as orange and Ser 66 as yellow spheres in Figure 6) increased Env-CT binding (in a pool down assay) and highlighted the importance of MA trimers for gp41-CT recognition. Disrupting this trimerization interface with small molecules or peptides could, therefore, actively interfere with the MA trimerization and hexameric Gag structures and possibly with Env incorporation and virus assembly.

A recent solution NMR structure of the gp41-CT revealed an unstructured N-terminal portion and a membrane-bound amphipathic helical region divided into three domains known as lentivirus lytic peptides, LLP2, LLP3, and LLP1 [74]. This structure can serve as a surrogate to identify a minimal binding region of this gp41-CT to MA. Peptides or small molecules mimicking this interaction could also potentially disrupt Env incorporation resulting in viruses devoid of Env, and therefore, noninfectious HIV-1 virus particles.

## 5. Capsid (CA, p24)

**Figure 7:**
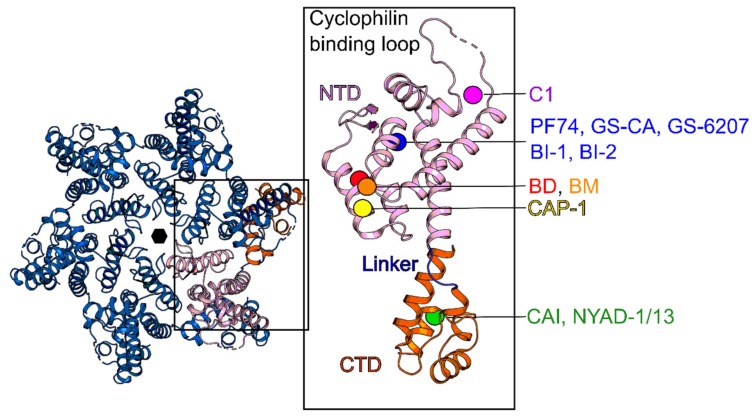
CA Inhibitors and binding site locations. CA is depicted in the context of a hexamer (**left**), and inhibitor/peptide-binding site is highlighted in the monomer (**right**). PDB code: 6ES8.

As a structural component of HIV-1, the capsid (CA) protein is responsible for the morphology of the immature Gag and the mature viral core with its characteristic conical structure. Within HIV-1 subtypes, CA is one of the most conserved proteins, and mutations are not well tolerated [75]. These characteristics make CA a highly attractive target for inhibitor design. Structurally, the CA protein is divided by a flexible interdomain linker into two domains, an N-terminal domain (NTD) and a C-terminal domain (CTD) (Figure 7) [76]. The CTD is the central driving unit during Gag oligomerization, and the NTD encodes a Pro-rich loop crucial for binding to cyclophilins [77,78]. During viral maturation (late-phase) and translocation of the immature Gag precursor (Pr55Gag) to host cell membranes, CA-dependent hexagonal lattice structures can be observed [79,80]. Further processing by the virally encoded protease forms the mature CA hexagonal conical structure with pentameric rings at both ends of the cone to close off the cone [81]. Hexamers and pentamers are stabilized by NTD-NTD interactions and intermolecular NTD-CTD interactions, while the extended hexameric lattice is connected via CTD-CTD interactions [82]. Core stability is essential for viral replication, and due to its high conservation, mutations that stabilize or destabilize the core result in altered infectivity [83]. The viral core undergoes uncoating by interacting with various host cell proteins such as dyneins, the kinesin-1 adaptor FEZ1, and transportin-1; however, also partly dissembled structures can be found at the nuclear pore gates [84,85,86,87,88]. Host cell restriction factors have also been shown to recognize CA such as MxB [89,90], TRIM5a [91] and TRIMCyp [92], which accelerates uncoating and release of viral DNA, which can be sensed by other restriction factors such as the cyclic guanosine monophosphate–adenosine monophosphate synthase (cGAS) [93]. The CA domain in the Gag protein is crucial for multiple steps in the viral replication cycle, and inhibitors can target both early and late-stage processes by stabilizing or destabilizing core structures. This involvement in numerous steps throughout the lifecycle and its high conservation resulting in a high barrier for resistance mutations makes CA an attractive target. During the last years, small molecules and peptide-based antivirals have been designed that disrupt CA-CA interactions in the immature Gag lattice, the mature core, or both, and the following section will describe the evolution of inhibitor design that target different bindings sites of the HIV-1 CA protein.

**Figure 8:**
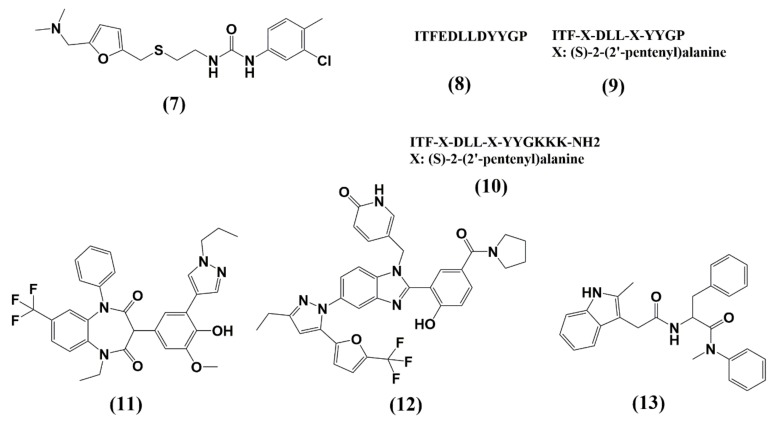
Structure of CA inhibitors discussed in this review. (**7**) CAP-1; (**8**) CAI; (**9**) NYAD-1; (**10**) NYAD-13; (**11**) BD-1; (**12**) BM-1; (**13**) PF74.

Tang and colleagues developed the first small molecule targeting the CA protein in 2003 via a computational screen [94]. CAP-1 (7) inhibits HIV-1 in a dose-dependent manner (Infectivity reduced by 95% at 100 µM of CAP-1), and virus particles in the presence of CAP-1 showed abnormal core morphologies, consistent with inhibited CA–CA interactions during virus assembly and maturation. CAP-1 acts during the late-stage and defective core structures resulted in noninfectious particles. NMR and X-ray crystallography revealed that CAP-1 binds and alters the conformation of the NTD by displacing Phe32 and providing a hydrophobic pocket for an aromatic ring from CAP-1, thus leading to disruption of intermolecular NTD-CTD interactions within a hexamer ( Figure 7; Figure 8, Table 1) [95]. Besides classical small molecule compounds, a 12-mer peptide CA inhibitor (CAI (8)) was discovered in 2005 using a phage display library screen [96]. CAI binds in a hydrophobic CA dimerization interface and inhibits CA self-association in vitro. As with many peptides, CAI cannot penetrate the cell membrane, limiting its application as an antiviral agent. However, hydrocarbon stapling of the peptide resulted in the more stable alpha-helical peptides NYAD-1 (9) (IC_50_ = 4–15 µM) and NYAD-13 (10) [97,98]. Those peptides bind at the same site as CAI but display enhanced affinity, increased cell permeability, and inhibit the replication of numerous laboratory and clinical HIV-1 strains (Figure 7 and Figure 8, Table 1).

Other classes of CA-assembly inhibitors are the benzodiazepiene (BD) (11), and benzimidazole (BM) (12) compounds with EC_50_ < 100 nM. These compounds bind similar to CAP-1 at the tip of the NTD (Figure 7 and Figure 8, Table 1). However, in contrast to CAP-1, BDs inhibit the assembly of the immature Gag lattice, preventing virus production while BMs disrupt virus maturation and reduce infectivity [99,100].

The interprotomer pocket composed of regions from NTD and CTD is the binding site of maybe the best known CA inhibitor to date, the Pfizer compound PF-3450074 (13) (also known as PF74), which inhibits HIV-1 replication at submicromolar potencies (EC_50_ = 8–640 nM) [101,102]. PF74 binds at the NTD-CTD subunit interface and occupies a similar pocket used by the host proteins CPFS6 and Nup153, two nuclear import factors known to enhance infectivity by increasing nuclear import and integration (Figure 7 and Figure 8, Table 1) [103,104,105]. PF74 stabilizes the CA core structure upon infection, which inhibits the uncoating process and, subsequently, HIV-1 reverse transcription [101,106,107] in the early stage and destabilizes CA in the late-stage causing aberrant virus morphologies that do not undergo maturation. PF74, however, suffers from extremely poor drug-like properties due to its peptidic nature, most notably its poor metabolic stability, which limits its clinical utility. During the last decade, numerous research groups, therefore, have tried to improve metabolic stability and potency of PF74 [107,108,109,110].

**Figure 9:**
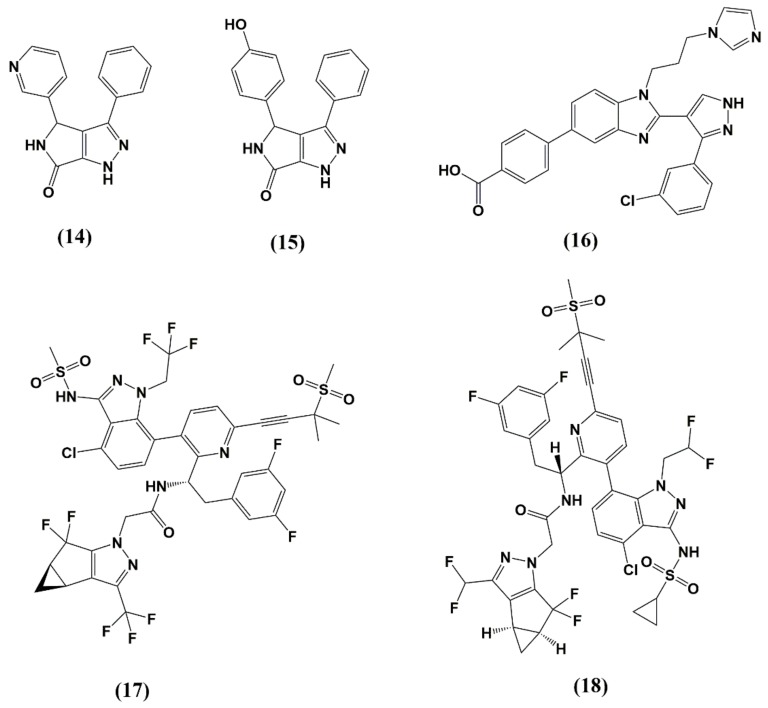
Structure of CA inhibitors discussed in this review. (**14**) BD-1; (**15**) BM-1; (**16**) C1; (**17**) GS-CA1; (**18**) GS-6207.

The pyrrolopyrazolones BI-1 (14) (EC_50_ = 8.2 µM) and BI-2 (15) (EC_50_ = 1.8 µM), discovered by Boehringer Ingelheim occupy the same binding site as PF74, and show a similar stabilizing effect on the CA lattice [111] and seems to compete with CPSF6 and Nup153 for CA binding, suggesting disruption of nuclear import (Figure 7 and Figure 9, Table 1) [104,112].

Close to the CypA binding loop (NTD), C1, another novel inhibitor, was found to bind (Figure 7 and Figure 9, Table 1) (IC_50_ = 57 µM) [113]. C1 also inhibits CA assembly in vitro. However, the exact mechanism of action is still under debate, but inhibition of HIV-1 replication might be achieved by acting at the late stage and disrupting the mature viral capsid [114]. Recently, using a novel time-resolved fluorescence resonance energy transfer (TR-FRET) assay screening, a 1280 compound library, Ebselen, was discovered [115]. Ebselen inhibits CA dimerization in vitro and inhibits HIV-1 replication with an EC_50_ of 3.37 µM without affecting particle assembly and maturation, indicating an early stage effect. Ebselen also inhibits reverse transcription and impairs uncoating by CA stabilization according to a cell fractionation assay and NMR studies. However, further studies are needed to confirm CA as the actual target through which these antiviral effects are mediated.

The CA inhibitors described thus far have not passed preclinical testing due to low potency or suboptimal drug-like properties; however, in 2017, a new CA inhibitor was described. GS-CA1 exhibits high antiviral potency in human peripheral blood mononuclear cells (EC_50_ = 140 pM) and broad-spectrum inhibition against all major HIV-1 clades [116]. In multiple preclinical species, the low systematic drug clearance and long half-life (7.2–18.7 h) combined with low aqueous solubility imply a long-acting potential [117]. In vitro resistance mutations were also identified; however, none of the five identified mutations are currently present in 132 analyzed circulating strains. Most recently, a derivative of GS-CA1, GS-6207, was presented at the Conference on Retroviruses and Opportunistic Infections in Seattle, Washington. GS-6207 has a potent and selective antiviral activity in MT-4 cells (EC_50_ = 100 pM, CC_50_ = 27 µM) and a mean EC_50_ of 50 pM in 23 clinical isolates [118]. GS-6207 is a promising CA inhibitor that stabilizes the HIV-1 capsid and disturbs the formation of the mature core. Molecular docking studies of both GS-CA compounds predict binding in the pocket that is shared by PF74 and host cell factors such as CPSF6 and Nup153 within the NTD-CTD intersubunit interface (Figure 7 and Figure 9, Table 1) [119]. GS-CA1 and GS-6207 are born out of the PF74 and share the same polyphenyl core and are believed to possess a similar but more potent mechanism of action compared to PF74.

GS-6207 demonstrated in vitro low solubility, high lipophilicity, and high metabolic stability in human hepatocyte assays, and in multiple animal models, one single subcutaneous application showed low clearance, moderate volume distributions, and long half-life (15–38 h). To date, GS-6207 is the only CA inhibitor that has entered the clinical phase; and Phase I and Ib randomized studies to establish safety, tolerability, and pharmacokinetics show promising results for the first long-acting CA inhibitor. However, future Phase 2 and 3 clinical trials are crucial and will provide important long-term safety and efficacy data of this CA inhibitor.

## 6. Nucleocapsid (NC, NCp7)

**Figure 10:**
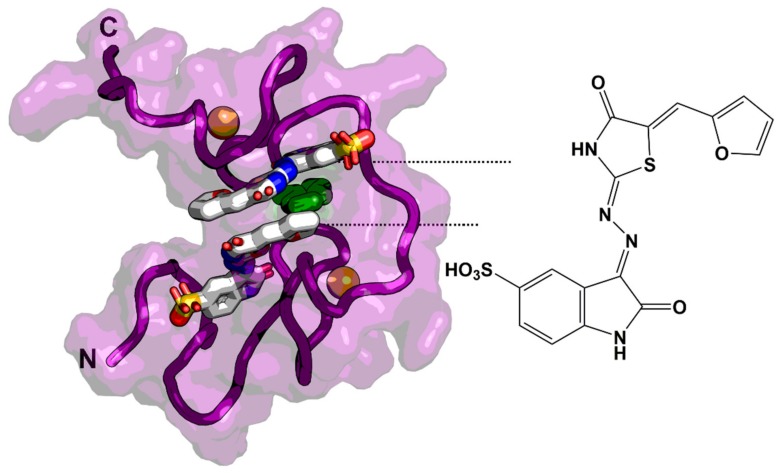
NMR complex structure with an NCI (compound 3) (2:1 stoichiometry). The NCI binds within a hydrophobic pocket and is stabilized by π-π stacking with Trp37 (highlighted in green). The model was derived from PDB code: 2M3Z. Zn^2+^ ions are represented as orange balls in both zinc fingers.

The nucleocapsid (NC) protein is a small (7 kDa) basic protein, also known as NCp7, and is located at the C-terminal portion of the Gag polyprotein [120]. NC binds nucleic acids via its two CCHC motif zinc fingers that are highly conserved among retroviruses, and nucleic acid binding promotes Gag oligomerization (Figure 10) [20,121]. The NC domain is, therefore, crucial in recruiting viral genomic RNA into the virus particles, explicitly recognizing the packaging signal in the genomic RNA. In addition to Gag assembly and nucleic acid binding, NC also facilitates post-entry events such as reverse transcription [122,123]. NC, therefore, contributes to HIV-1 replication mainly by its chaperone functions via specific interactions with various forms of nucleic acids. Due to its involvement in reverse transcription and integration, single point mutations can lead to fully non-infectious viruses highlighting NCs importance in the HIV-1 life cycle [124,125]. Given this multifunctional role of this small but crucial protein, several inhibitors have been designed over the last years, including zinc-ejectors, non-zinc ejecting NC binders, nucleic acid intercalators, peptidomimetics, and RNA aptamers [126].

### 6.1. Zinc-Ejectors

**Figure 11:**
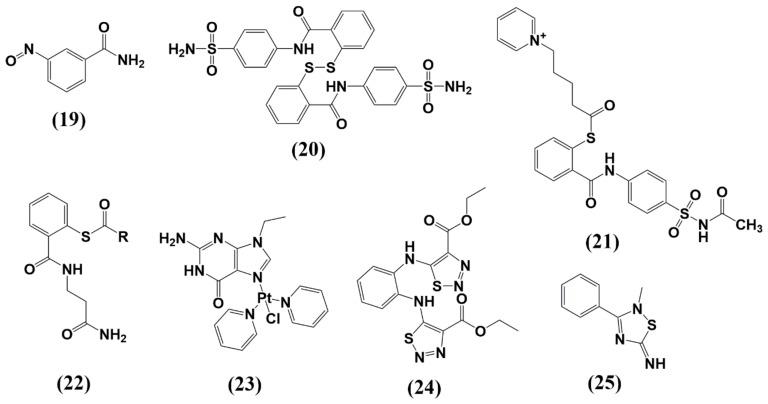
Structure of NC inhibitors discussed in this review. (**19**) NOBA; (**20**) DIBA; (**21**) PATE; (**22**) SAMT; (**23**) trans-chlorobispyridine (9-ethylguanine)platinum(II); (**24**) NVO38; (**25**) WDO-217.

**Figure 12:**
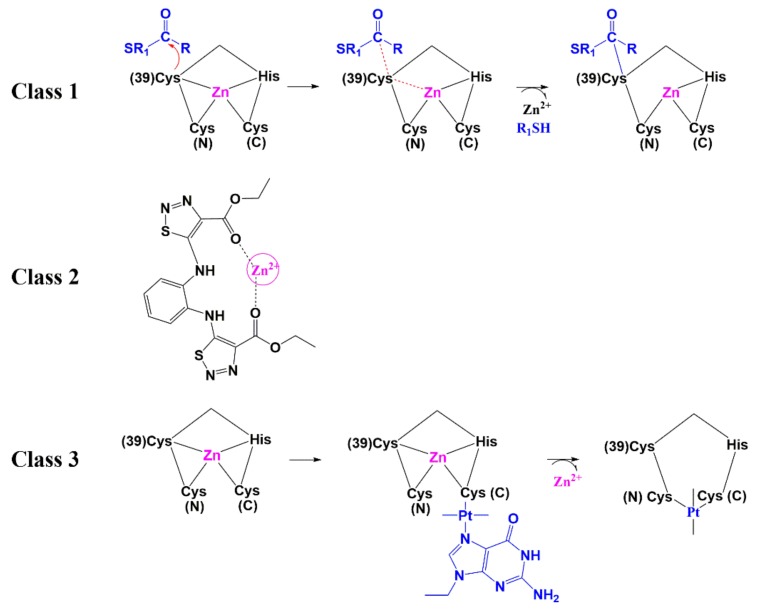
Zinc ejecting mechanisms: Class 1) Electrophilic attack of the zinc fingers. The nucleophilic attack of the cysteine 39 results in the formation of a thioester by SAMTs. This covalent linkage stimulates the reaction of additional reactants with the other Cys residues of the same motif and leads to reduction in Zn^2+^ affinity of the zinc finger and finally zinc ejection and NC unfolding [131]. Class 2) Zinc chelation by the two carbonyl oxygens of the ester from NVO38 [133]. Class 3) Covalent binding of Cys residues by platinum (Pt) as represented by *trans*-chlorobispyridine(9-ethylguanine)platinum(II) [137]. Cys (N) and Cys (C) represent N-terminal and C-terminal cysteines in the zinc finger.

Given the importance of zinc ions to fold NC into its functional form, zinc ejectors are among the first developed NC inhibitors. The ejectors were found to induce NC unfolding and total loss of HIV-1 infectivity [127,128]. Most of them display high antiviral activity with low resistance mutation rates; however, their systematic application was limited due to their cytotoxic effects. This includes compounds such as 3-nitrosobenzamide (NOBA) from the C-nitroso-class [127], 2,2-dithiobisbenzamide disulfides (DIBA) [129], pyridinioalkanoyl thioesters (PATE) [130], Sacyl- 2 mercaptobenzamide thioesters (SAMT) [131], and transchlorobispyridine (9-ethylguanine) platinum(II) [132]. The most recent NC inhibitors from the ejector class are N,N′-bis(4-ethoxycarbonyl-1,2,3- thiadiazol-5-yl)benzene-1,2-diamine (NV038) [133] and 2-methyl-3-phenyl-2H-[1,2,4]thiazol-5-yideneamine (WDO-217) [134] (Figure 11 and Table 1). The mechanism by which the ejector targets the NC can be classified into three mechanisms: 1) electrophilic attack of the zinc fingers 2) zinc chelation, and 3) covalent binding of Cys residues by platinum (Pt) (Figure 12). Although both Cys residues are reactive, the distal (C-terminal) nucleophilic cysteine thiolate is the main target for an electrophilic attack, due to its higher accessibility [135,136]. The electrophilic attack is facilitated by intra- or intermolecular disulfide bond formation or acylation of cysteine and lysine residues. This is supported by the fact that the class 1 ejectors are prodrugs and acylated intracellularly prior to target recognition.

### 6.2. Small Molecules as Non-Zinc Ejectors

In addition to zinc ejectors, several non-covalent inhibitors (NCIs) were identified during the last decade. However, none of them are in preclinical or clinical development to date, highlighting the challenges to target NC. Given the higher specificity of NCIs to NC, this class is a promising pharmaceutical goal to discover new and less cytotoxic compounds that compete with NC for RNA/DNA or other interaction partners. A more detailed evaluation of these inhibitors is reviewed in [126,138]. We, therefore, focus on a few recent developments in the following section (Table 1).

In a high-throughput fluorescence polarization assay, Breuer et al. discovered two compounds that specifically bind to NC with nanomolar affinity and inhibit HIV-1_NL4-3_ with EC_50_ values of 0.32 and 3.5 µM [139]. Boehringer Ingelheim described in 2013 another NCI (compound 3) that disrupts the interaction of NC with RNA and inhibits HIV-1 replication with low-micromolar EC_50_s [140]. Due to its high flexibility, X-ray crystallography is challenging, and most of NC structures are solved by NMR. The NMR structure of HIV-1 NC in complex with the Boehringer Ingelheim inhibitor provided important structural insights into RNA displacement (Figure 10).

The NCI binds in a 2:1 ratio in a hydrophobic pocket, providing π-π stacking interactions with Trp37, thus mimicking the guanosine base of the NC nucleic acid binder. Facilitated by the NMR high-resolution structure, rational optimization in silico resulted in the generation of AN3 (2-amino-4-phenylthiazole NCI), an efficient, non-toxic NCI with antiviral activity in cells [141]. Taken together, these structures provided important insights and supported the drugability of NC towards more improved and efficient drug-like NCIs. In a recent study, a new inhibitor was identified; A1752 shows antiviral activity with an IC_50_ of around 1 µM [142]. A1752 recognized NC directly, thereby inhibiting specifically its chaperone function, including Psi RNA dimerization and complementary trans-activation response element (cTAR) DNA destabilization. In addition, A1752 disrupted proper Gag processing and generated noninfectious viral particles with uncoating and reverse transcription defects in infected cells. These few examples highlight the possibility and future need to target a highly flexible multifunctional key player in HIV-1 replication. The genetic barrier for resistance mutations is extremely high and not without consequences for virus replication and provides a strong argument for future NC inhibitor development.

## 7. Late Domains (P6)

HIV-1 and many other non-retroviral enveloped viruses utilize the host cell machinery for virus particle budding, scission, and release at the plasma membrane. One of the main pathways hijacked by the virus involves the ESCRT machinery comprising four complexes, namely ESCRT-0, I, II, III, including other host adaptors associated with this machinery. One key player is the cellular protein Tsg101 (tumor susceptibility gene 101) as part of ESCRT-I. Tsg101 is recruited to viral assembly sites via the late domain of the Gag polyprotein, also known as p6. The directed particle release is accomplished via a Pro-Thr-Ala-Pro (PTAP) motif in p6 that serves as a docking site for Tsg101 [143,144,145]. This interaction is critical for HIV release, highlighted by the high conservation within the PTAP motif [146]. Additionally, the Tyr-Pro-Xn-Leu motif in p6 (YPXnL with X is any residue and n can vary from 1 to 4 amino acids) binds to the ESCRT-associated factor Alix (ALG-2 (apoptosis-linked gene 2-interacting protein X).

**Figure 13:**
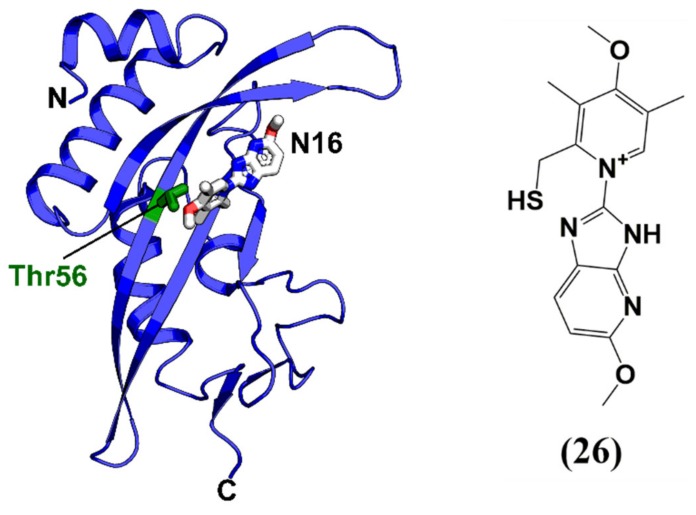
Solution NMR structure of Tsg101 complexed with N16. N16 binds in a hydrophobic groove defined by Thr56 (in green), similar to other peptide-based Tsg101 inhibitors. (**26**) N16. PDB code: 5VKG.

The majority of HIV-1 budding antagonists are focused on the disruption of the Tsg101 or Alix interface [147,148,149]. Liu and colleagues in 2006 designed and tested N-substituted glycine variations of PTAP by incorporating hydrazine amides (peptoid hydrazones) [150]. The best n-butyl containing peptoid hydrazine (K_D_ = 9.8 µM) displayed a five-fold increased affinity towards Tsg101 compared to wild-type PTAP peptide. In another attempt to identify peptides blocking the p6-Tsg101 interaction, a bacterial reverse two-hybrid system was utilized to screen a cyclic peptide library of 3.2 × 10^6^ members, and the best peptide (cyclic peptide 11) inhibited the production of virus-like particles (VLPs) of cultured human cells with an IC_50_ of 7 µM (Table 1) [151]. Besides the development of peptides to disrupt the p6-Tsg101 interaction, a recent study identified via a high-throughput screen of a small molecule library, two small molecules F15 (esomeprazole) and N16 (tenatoprazole) that are capable of binding to the UEV (ubiquitin E2 variant) domain of Tsg101 [152]. F15 is currently used for indications of heartburn (or indigestion), and N16 was undergoing phase I clinical trials in July 2016 as a proton pump inhibitor as a potential treatment of reflux oesophagitis. Both compounds could reduce Gag assembly and virus production in vitro, highlighting the possibility of using small molecules like F15 and N16 to interfere with a previously unrecognized Tsg101 contribution to budding. The solution NMR complex structure of N16 together with Tsg101 also provides a rational and future perspective for the improvement of Tsg101 inhibitors (Figure 13 and Table 1).

## 8. Conclusions

Despite the incredible success of AIDS treatment during the last years with current ART therapies, resistance mutations, and the accumulation of severe side effects is an enormous challenge that continually needs to be addressed. In this review, we highlight the importance of the HIV-1 Gag polyprotein as the master coordinator of HIV-1 assembly and maturation. The individual Gag domains play crucial roles in the HIV-1 replication cycle and are therefore appropriate but also challenging targets of inhibitor development. However, recent positive developments in targeting the Gag domains such as the capsid protein with highly potent and potentially long-acting inhibitors, as well as the exploration and successful targeting of challenging HIV-1 proteins such as the matrix protein, are very encouraging in the fight against the AIDS pandemic. Such new inhibitors can also serve as novel probes to dissect and better understand HIV-1 biology.

## Figures and Tables

**Table 1:** HIV-1 Gag inhibitors from pre-clinical and clinical development.

Protease (PR) and Maturation Inhibitors (MI)	Target and Binding Site	Mechanism of Action	Antiviral Potency (IC_50_)	CC_50_	Clinical Status
Bevirimat (BVM)	CA-SP1 junction site	Stabilizes six-helix bundle in CA hexamer and prevents CA-SP1 cleavage	~10 nM	~25 μM	Failed in phase IIb due to resistance mutations in CA-SP1
PF-46396	CA-SP1 junction site	Implications for Gag assembly, release and virus replication	0.005–7 μM (PBMCs)	17 μM (PBMCs)	Not entered
GSK3532795	CA-SP1 junction site	Late-stage inhibition of CA-SP1 cleavage	21 nM	2.3 to > 15 μM	Post phase IIb termination due to high rates of adverse gastrointestinal events, and frequency of treatment-emergent nucleoside reverse transcriptase inhibitor (NRTI) resistance
**Matrix (MA) Inhibitors**	**Target and Binding Site**	**Mechanism of Action**	**Antiviral Potency (IC_50_)**	**CC_50_**	**Clinical Status**
(Thiadiazolane class) e.g., TD2	MA RNA binding site	RNA displacement	1–5 μM	5-20 μM	Not entered
Compound 7 and 14	MA PI[4,5]P_2_ binding site	PI[4,5]P_2_ displacement	7.5–15.6 µM (group M isolates)	Compound 7 and 14 = >100 µM (PBMCs); compound 7 = >1 mM (293T cells)	Not entered
**Capsid (CA) Inhibitor**	**Target and Binding Site**	**Mechanism of Action**	A**ntiviral Potency (IC_50_)**	**CC_50_**	**Clinical Status**
CAP-1	NTD	Blocks CA self-association in late events	EC_95_ ≈ 100 μM	>100 μM	Not entered
Peptide Inhibitors (CAI, NYAD-1)	CTD	Blocks assembly of immature and mature-like particles	N.D. (CAI) = 4.29–21.6 μM (NYAD-1 PBMCs)	N.D. (CAI) N.D. (NYAD-1)	Not entered
BD-1	NTD	Blocks CA assembly	70 ± 30 nM	>28 μM	Not entered
BM-1	NTD	Blocks HIV-1 maturation	62 ± 23 nM	>20 μM	Not entered
PF74	NTD-CTD	Stabilizes CA core in early-stage and inhibits reverse transcription. Distorts CA lattice in the late stage, causing aberrant virus morphology that does not undergo maturation	80–640 nM (PBMCs)	>10 μM (PBMCs)	Not entered
BI Compounds (BI-1, BI-2)	NTD	Destabilizes HIV-1 capsid by interfering in early and late events	7.5 ± 2.1 μM (BI-1) 1.4 ± 0.66 μM (BI-2)	>91 μM (BI-1) >76 μM (BI-2)	Not entered
C1	NTD	Inhibits HIV-1 replication in late events by disrupting the assembly of the mature capsid	57 µM	N.D.	Not entered
Ebselen	Undetermined	Reverse transcription inhibition and impaired uncoating	3.37 µM	>30 μM (PBMCs)	Not entered
GS-CA1 and GS-6207	NTD-CTD	Most likely, stabilizes CA core in early-stage and inhibits reverse transcription. Probably, distorts CA lattice in the late stage, causing aberrant virus morphology that does not undergo maturation	140 pM (GS-CA1, PBMCs) 100 pM (GS-6207, MT-4 cells)	27 µM (GS-6207)	Phase 1b (GS-6207)
**Nucleocapsid (NC) Inhibitors**	**Target and Binding Site**	**Mechanism of Action**	**Antiviral Potency (IC_50_)**	**CC_50_**	**Clinical Status**
NOBA	Zinc finger	Class 1 - electrophilic attack of the zinc fingers	N.D.	10.6 µM	Not entered
DIBA-1	Zinc finger	Class 1 - electrophilic attack of the zinc fingers	2.3 µM	>200 µM	Not entered
PATE-45	Zinc finger	Class 1 - electrophilic attack of the zinc fingers	6.2 µM	>316 µM	Not entered
SAMT-19	Zinc finger	Class 1 - electrophilic attack of the zinc fingers	2.9 µM	461 µM	Not entered
[SP-4-2]-[PtCl(NH_3_) (quin)(9-EtGH)]	Zinc finger	Class 3 - covalent binding of Cys residues by platinum	41.9 µM	>200 µM	Not entered
NVO38	Zinc finger	Class 2 - zinc chelation	17 µM	>300 µM	Not entered
WDO-217	Zinc finger	Class 1 - electrophilic attack of the zinc fingers	7.9 µM	72 µM	Not entered
Compound 3	Two molecules bind each zinc knuckle of the NC	Mimicking the guanosine base found in many reported NC complex structures	0.95 μM (NC-oligonucleotide binding assay)	N.D.	Not entered
A1752	NC	Inhibits NC-mediated dimerization of Psi RNA and cTAR DNA destabilization. Inhibits also proper Gag processing	~1 µM	>50 μM	Not entered
**Late domains (P6) Inhibitors**	**Target and Binding Site**	**Mechanism of Action**	**Antiviral Potency (IC_50_)**	**CC_50_**	**Clinical Status**
Cyclic peptide 11	P6-Tsg101 interface	Blocking the p6-Tsg101 interaction	7 µM	N.D.	Not entered
N16	Ubiquitin E2 variant domain of Tsg101	Reduces Gag assembly and virus production in vitro	EC_50_ between 25 and 50 μM (p24 ELISA)	>50 μM	Phase I as a proton pump inhibitor

N.D. Not determined; half-maximal inhibitory concentration (IC_50_) is represented from cell-based assays if not other stated in the table; half-maximal cytotoxic concentration (CC_50_). References and structures for the individual inhibitors can be found within the corresponding Gag domain sections.
